# Case report: Unique ultrasound feature of thyroid metastases in occult breast cancer

**DOI:** 10.3389/fonc.2022.970286

**Published:** 2022-10-03

**Authors:** Kaining Zhang, Yong Yu, Yichen Zang, Hua Xu, Beibei Lv, Qian Wang

**Affiliations:** ^1^ Department of Ultrasound, Shandong Provincial Hospital, Shandong University, Jinan, China; ^2^ Department of Ultrasound, Shandong Provincial Hospital affiliated to Shandong First Medical University, Jinan, China; ^3^ Department of Abdominal Ultrasound, The Affiliated Hospital of Qingdao University, Qingdao, China; ^4^ Department of Infectious and Endemic Disease Control, Shizhong District Center for Disease Control and Prevention, Jinan, China; ^5^ Department of Pathology, Shandong Provincial Hospital Affiliated to Shandong University, Jinan, China

**Keywords:** thyroid metastases, occult breast cancer (OBC), ultrasound, thyroiditis - ultrasound, case report

## Abstract

Occult breast cancer is an uncommon type of breast cancer. Metastases of occult breast cancer to other tissues are rather rare. We present a rare case of thyroid metastases in a 46-year-old woman who underwent occult breast cancer. The first ultrasound (US) examination of the thyroid showed that the left lobe was enlarged but had normal thyroid function. At first, this case was misdiagnosed as thyroiditis based on the thyroid US features. However, the cytological and histological results showed that nests of the neoplastic cells were found. Further immunohistochemistry results confirmed that these neoplasms were derived from breast tissue. Analysis using the successive US scans revealed that the sizes and echo of the thyroid repeatedly changed after the radiotherapy and chemotherapy treatment. To our knowledge, this is the first reported case of occult breast carcinoma presenting with thyroid metastases. This case can easily be misdiagnosed as thyroiditis due to the metastasis area not manifesting as regular suspicious nodules or diffused punctate calcifications.

## Introduction

Occult breast cancer (OBC) is a special type of breast cancer which was first reported and described as “cancerous axillary glands with non-demonstrable cancer of the mamma” in 1907 (1). At present, OBC is histologically diagnosed in the axillary lymph nodes through biopsy, but no clinical or radiographic methods, such as mammography, ultrasonography (US), or magnetic resonance imaging (MRI), have been found to be suitable (2). The incidence of OBC among all types of breast cancer is about 0.1%–1% (3).

Due to its rarity and lack of primary focus within the breast, OBC cannot be diagnosed at an early stage. If OBC cannot be treated early enough, metastasis can occur in a majority of tissues, such as the gastrointestinal tract (4) and skin (5). Moreover, the prognosis of OBC is still debatable. The 10-year overall survival of OBC in different reports varies from 45% to nearly 70%. The thyroid gland is a relatively infrequent site of metastasis. Herein, we report on a case of a 46-year-old female patient with metastatic OBC in the thyroid.

## Case description

A 46-year-old woman was referred to the Radiation Oncology Department of Shandong Provincial Hospital (Jinan, Shandong) on 9 December 2015 and presented with a hardened mass in the right axilla. Ultrasound examination showed that the right axillary lymph nodes were swollen. No primary cancers were found in the right breast during the ultrasound examination and further mammography. A US-guided biopsy of the swollen lymph node in the right axilla confirmed metastases from the breast. This patient was treated with four cycles of neoadjuvant chemotherapy (docetaxel 120 mg d1 + nedaplatin 40 mg d1–3) before undergoing modified radical mastectomy half a year after the biopsy. Postoperative pathological analysis revealed that there were no primary tumors in the breast tissue while metastases in the lymph node were confirmed. The patient received radiotherapy and chemotherapy treatment after the operation.

**Figure 1:**
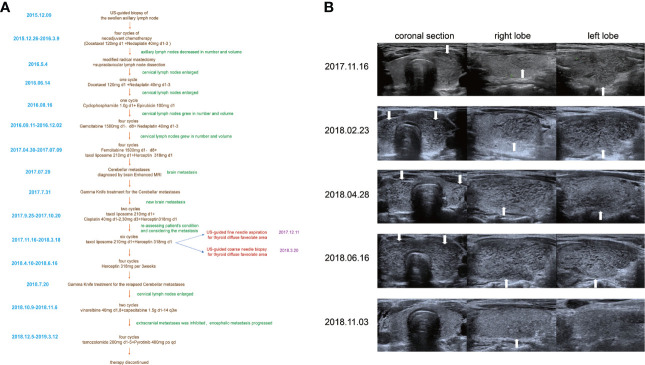
**(A)** The timeline of the whole management course of the patient with occult breast cancer. **(B)** One-year follow-up US features with thyroid metastases from her occult breast cancer (arrows show the metastasis areas which manifested as diffuse faveolate changes in the US).

The patient was treated with several cycles of chemotherapy, and although the chemotherapy regime was changed repeatedly, tumor progression was not inhabited. Cerebellar metastasis was diagnosed during the chemotherapy treatment (**Figure 1A**). Goiter was found in the regular computed tomography scan performed in November 2017. Then, the US of the thyroid was performed to further check the accuracy of thyroid manifestation. The US showed that the left lobe of the thyroid was enlarged and produced a diffused rough and faveolate echo, while the right lobe was rather homogeneous. No nodules were found in the thyroid. The patient was first diagnosed with subacute thyroiditis due to the non-uniformity between different lobes. However, the thyroid function was normal (free T3 5.09 pmol/l (3.5–6.5), free T4 14.8 pmol/l (11.5–22.7), thyrotropin 2.746 μIU/ml (0.55–4.78)). The thyroid-related antibody filters also within the normal range (thyroglobulin (TG) antibodies 23.90 IU/ml (0–60), thyroid peroxidase (TPO) antibodies 30.90 IU/ml (0–60), thyrotropin antibodies <0.30 IU/l (0–1.58)) Another US examination was performed on the patient after 3 months. Interestingly, the US showed that both lobes were enlarged and the echo of both lobes was diffuse faveolate.

**Figure 2:**
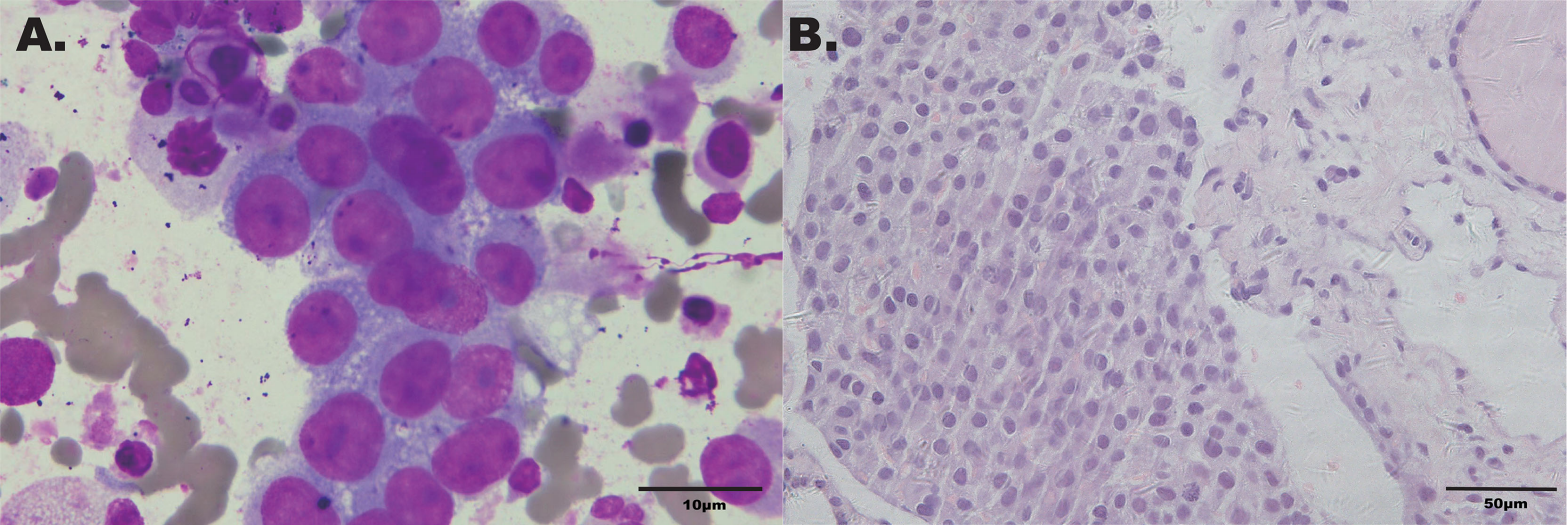
**(A)** Cytology smear of the thyroid faveolate echo area in the US (Wright–Giemsa ×1,000). **(B)** Histochemical staining of the biopsy specimen from thyroid (hematoxylin–eosin stain ×400).

**Figure 3:**
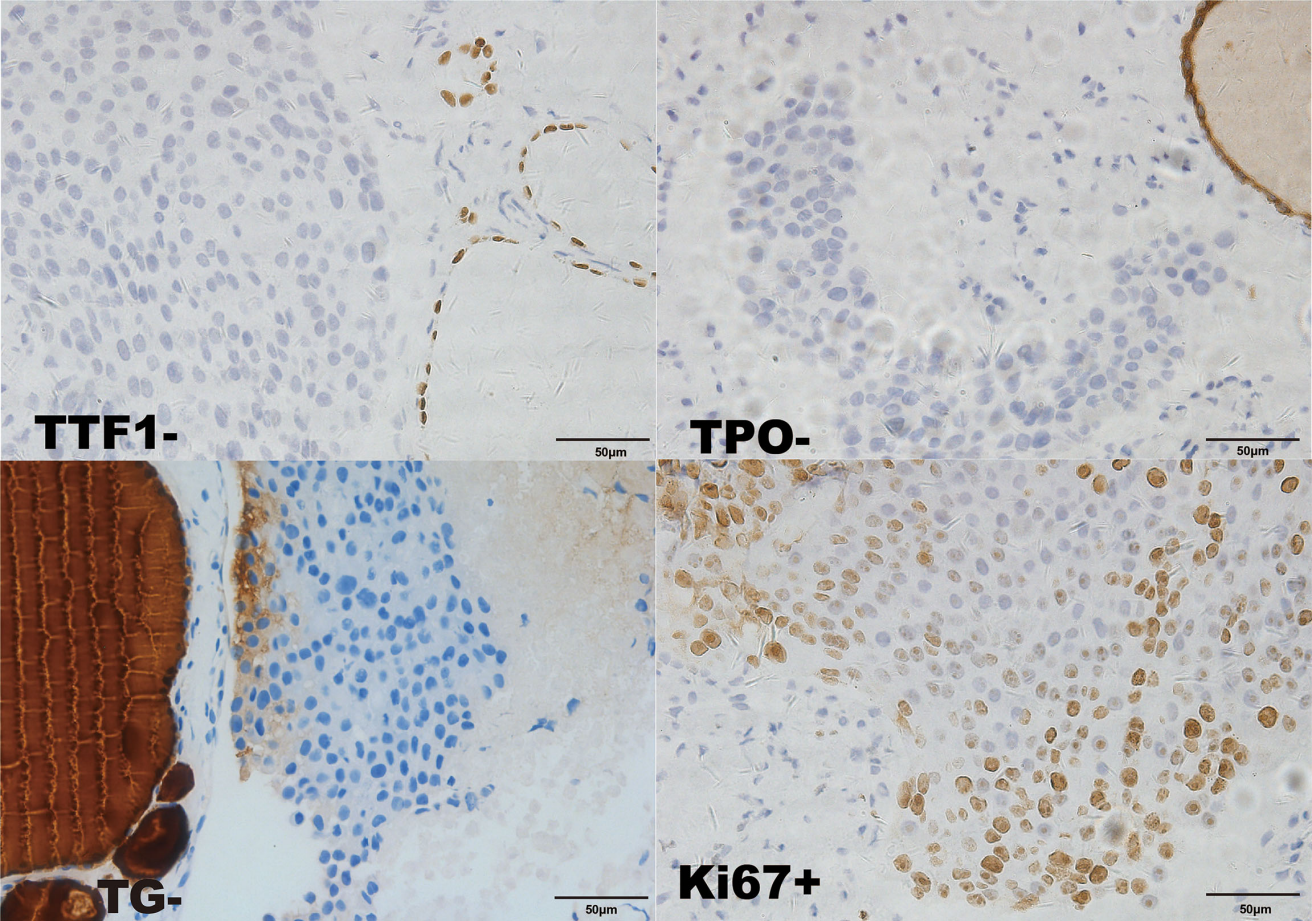
Immunohistochemical staining of the biopsy specimen from the thyroid (×400). TTF1-; TPO-; TG-; GATA-3-.

**Figure 4:**
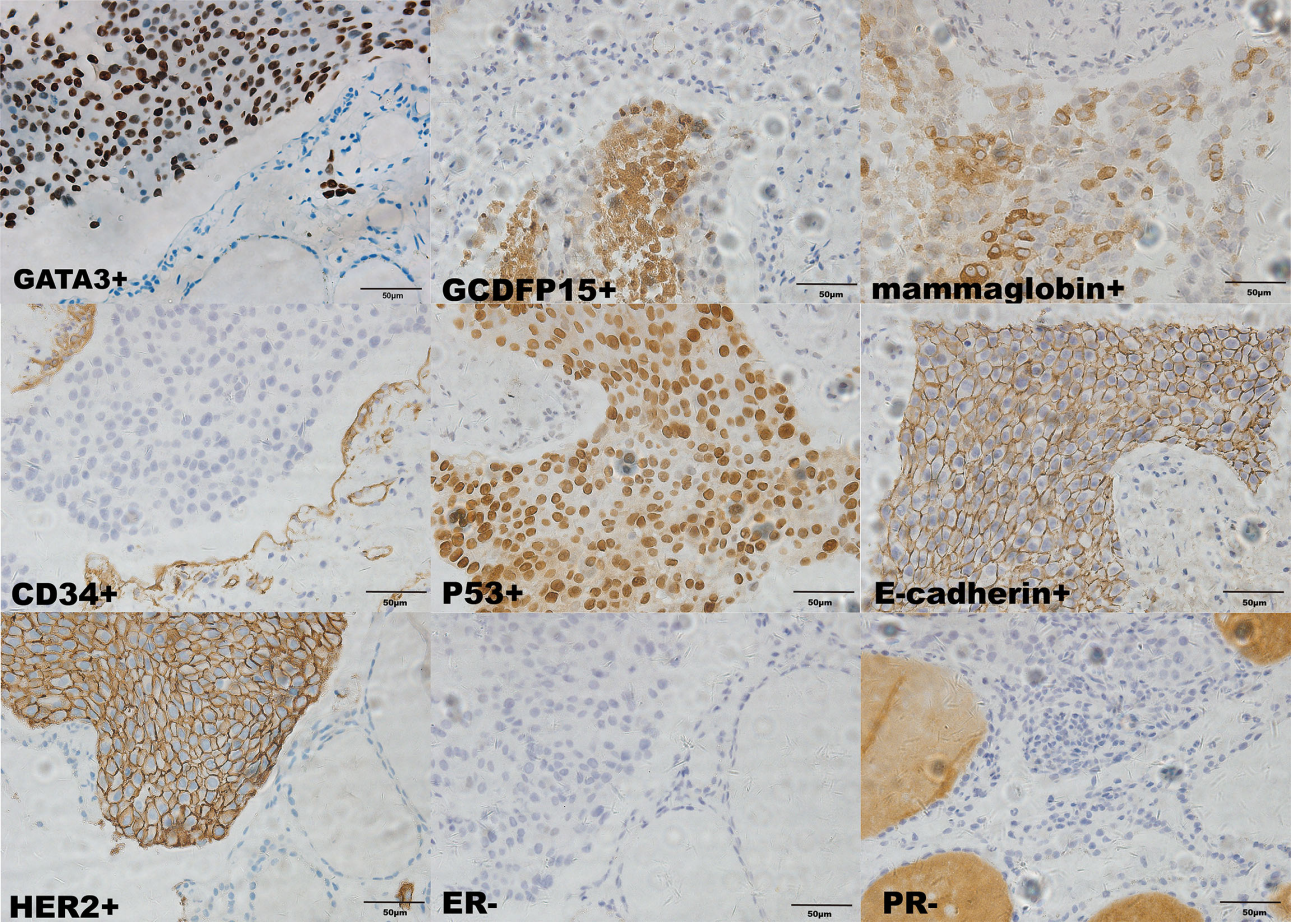
Immunohistochemical staining of the biopsy specimen from the thyroid (×400). Ki-67+ (60%); GCDFP15+; mammaglobin+; CD34+; P53+; E-cadherin+; HER2+; ER-; PR-.

Considering that laboratory tests of thyroid function did not correspond with the US findings and a history of occult breast carcinoma, a US-guided fine-needle aspiration was performed. The cytology showed a mass of tumor cells found in the smear (**Figure 2A**). To determine the original tissue of the tumor cells, a US-guided coarse needle biopsy was conducted. The histology showed that nests of neoplastic cells were found in the thyroid vessels (**Figure 2B**). Further, negative immunohistochemistry (IHC) results of thyroid transcription factor 1 (TTF1), TPO, and TG showed that the tumor cells were not derived from the thyroid (**Figure 3**). In addition, 60% of cells expressed the cell proliferation marker, Ki-67, reflecting a high degree of cell malignancy. Considering the history of occult breast cancer, IHC staining for biomarkers associated with breast cancer was performed. The positive staining of GATA3, gross cystic disease fluid protein 15 (GCDFP15), mammaglobin, CD34, P53, and E-cadherin confirmed that these neoplasms were infiltrating ductal carcinoma derived from the breast (**Figure 4**). The molecular subtype of our OBC case turned out to be human epidermal growth factor receptor 2 (HER2) positive due to the positive IHC result for HER2, negative estrogen receptor (ER), and negative progesterone receptor (PR) (**Figure 4**). The data on the antibodies of the IHC makers are summarized in the **Supplementary Table**. The patient was treated with six cycles of Taxol liposome 210 mg d1 + Herceptin 318 mg d1 and four cycles of Herceptin 318 mg per 3 weeks as we monitored the progress in the thyroid. The sizes and hypoechoic areas of the thyroid gland changed repeatedly during this process (**Figure 1B**). The size of the diffuse faveolate area of the thyroid was obviously shrunk as shown in the final US examination. Based on the poor physical condition of the patient after receiving more than 30 cycles of chemotherapy and the modified radical mastectomy, the total thyroidectomy was not performed and the therapy was discontinued due to encephalic metastasis progression.

## Discussion

OBC is defined as breast cancer that presents as nodal disease or distant metastasis in a patient without a history of prior breast cancer as nodal disease or distant metastasis without clinical, radiological, or pathological evidence of a primary lesion in the breast. OBC is rare, and its diagnosis is challenging. In the literature, there is a paucity of studies that are conclusive on the clinicopathological characteristics of the disease, as well as patient outcomes and disease management. The clinical and pathological characteristics of the patients were analyzed. The most common site of pathological diagnosis/metastasis was the axilla in 23 (74.2%) patients, followed by bone in three (9.7%), orbit in two (6.5%), and one each in the liver, lung, and brain (6). Moreover, the prognosis of OBC is still debatable. Although the 10-year overall survival of OBC in different reports has varied from 45% to nearly 70% (7), OBC patients with distant metastatic disease have a much worse prognosis, with a 5-year survival of 14.3% (6). Therefore, it is crucial to ascertain if an OBC has distant metastasis or not.

In the previous studies, among the subtypes of OBC, the luminal A subtype was the most common (27.40%) while HER-2 positive OBC merely accounted for about 6.20% (8). The subtypes of IHC of OBC showed no significant difference in prognosis. Pentheroudakis (9) theorized that patients with visceral metastases may be a distinct clinical subgroup with a similar molecular signature to breast carcinoma, which is different from patients with axillary nodal metastases. Our case had both axillary nodal and distant metastases, which may be one reason our patient had a poor prognosis. Additionally, the patient was treated with docetaxel and nedaplatin without Herceptin during neoadjuvant chemotherapy due to economic reasons. The patient was treated with Herceptin after 6 cycles of chemotherapy as cervical lymph nodes grew in number and volume. Failure to obtain Herceptin treatment earlier may have been a reason for multiple distant metastases.

The thyroid gland is a relatively infrequent site of metastasis. Although the thyroid is abundant in blood supply, the incidence of metastasis is low. The etiology and the mechanisms responsible for the low rate of thyroid metastasis are a quick arterial blood flow rate and a high concentration of oxygen and iodine, which may prevent metastatic deposits and inhibit the growth of malignant cells. Thyroid metastases of breast carcinoma are uncommon and usually develop in patients that experience a rare clinical event, too (10).

The thyroid is, in fact, very rarely involved even in metastatic OBC. To our knowledge, this is the first reported case of occult breast carcinoma presenting with thyroid metastasis. Not many studies have been conducted on the treatment of widely metastatic OBC. Similar to non‐OBC, systemic therapy, which includes chemotherapy, radiation therapy, and hormone therapy, is usually employed. Factors that must be considered in choosing the correct method of therapy include the number and sites of metastatic lesions as well as performance status (11). In light of the advanced breast cancer status of our patient, she was treated using docetaxel combined with nedaplatin. This regimen has proven efficacious for the treatment of ER-negative, HER2‐positive breast cancer. The size of the diffuse faveolate area in the thyroid was obviously reduced as observed in the last US examination after targeted therapy using Herceptin.

The metastasis to the thyroid from other organs is more likely to manifest as nodules with one or more specious features when observed under US examination (12, 13). The thyroid manifestation is unique even when compared with the thyroid metastasis from non-OBC, as described in the other studies (14). In our patient, the metastasis area in the thyroid in our case was not manifested to be nodules, which was rather misleading. The patient was misdiagnosed with thyroiditis several times. The hypoechoic areas in the gland reduced in size after the antitumor treatment which was thought to indicate that the lesions in the thyroid may be associated with OBC (**Figure 1B**). When dealing with short-term US manifestation changes, and especially in cases with a history of occult breast carcinoma, metastases from breast carcinoma should be suspected.

## Conclusion

Distant metastasis may cause a poor diagnosis due to not being treated in time. OBC patients must undergo a general checkup to exclude any distant metastasis. Thyroid US changes should be taken seriously and observed closely. Early diagnosis and aggressive management in the thyroid should be undertaken in the OBC patient for the atypical manifestations, similar to those that we observed in our OBC patient.

## Data availability statement

The raw data supporting the conclusions of this article will be made available by the authors, without undue reservation.

## Ethics statement

The studies involving human participants were reviewed and approved by Ethics Committee of Shandong Provincial Hospital. The patients/participants provided their written informed consent to participate in this study.

## Author contributions

KZ and YY conceived the idea for the article. QW managed the case and drafted the manuscript. YZ, HX, and BL revised the manuscript. KZ approved the final version of the manuscript. All authors contributed to the article and approved the submitted version.

## Funding

The work was supported by the Natural Science Foundation of Shandong Province (grant number ZR2021QH047 and ZR2021MH309) and the Clinical Science and Technology Innovation Development Program of Jinan. (grant number 202134036).

## Conflict of interest

The authors declare that the research was conducted in the absence of any commercial or financial relationships that could be construed as a potential conflict of interest.

## Publisher’s note

All claims expressed in this article are solely those of the authors and do not necessarily represent those of their affiliated organizations, or those of the publisher, the editors and the reviewers. Any product that may be evaluated in this article, or claim that may be made by its manufacturer, is not guaranteed or endorsed by the publisher.
