# Supplementary material for: Case report: Unique ultrasound feature of thyroid metastases in occult breast cancer
Source: Front Oncol. 2022 Oct 3;12:970286. doi: 10.3389/fonc.2022.970286 (PMC9575947; doi:10.3389/fonc.2022.970286)
Supplement: Supplementary file 1 [file Table_1.docx]

Table 1. Antibodies information in the Immunohistochemistry of 13 markers

| Marker | Ig Isotypes | clone number | dilution | positive location |
| --- | --- | --- | --- | --- |
| TTF1 | IgG1/κ | 8G7G3/1 | 1:200 | nucleus |
| TPO | IgG1 | AC25 | 1:100 | cytoplasm |
| TG | IgG1 | OTI8F2 | 1:500 | cytoplasm |
| Ki-67 | IgG2a | UMAB107 | 1:150 | nucleus |
| GATA3 | rabbit IgG | EP368 | 1:150 | nucleus |
| GCDFP15 | rabbit IgG | EP95 | 1:150 | cytoplasm |
| mammaglobin | IgG1 | 304-1A5 | 1:200 | cytoplasm |
| CD34 | IgG1 | 10C9 | 1:200 | cell membrane/ cytoplasm |
| P53 | IgG2b/κ | DO-7 | 1:200 | nucleus |
| E-cadherin | IgG1 | UMAB184 | 1:200 | cell membrane |
| HER2 | rabbit IgG | EP277 | 1:200 | nucleus |
| ER | IgG1 | pS2.1 | 1:150 | cytoplasm |
| PR | IgG1 | OTI2E2 | 1:150 | nucleus |

TTF1: thyroid transcription factor1; TPO: Thyroid peroxidase; TG: Thyroglobulin; GATA3: GATA binding protein 3; GCDFP15: Gross cystic disease fluid protein 15; CD34: Cluster of differentiation 34; P53: protein 53; HER2: Human epidermal growth factor receptor 2; ER: estrogen receptor; PR: progesterone receptor. All the bodies were acquired from Zhongshan Golden Bridge Biotechnology.
